# SOX18-enforced expression diverts hemogenic endothelium-derived progenitors from T towards NK lymphoid pathways

**DOI:** 10.1016/j.isci.2023.106621

**Published:** 2023-04-08

**Authors:** Ho Sun Jung, Kran Suknuntha, Yun Hee Kim, Peng Liu, Samuel T. Dettle, Divine Mensah Sedzro, Portia R. Smith, James A. Thomson, Irene M. Ong, Igor I. Slukvin

**Affiliations:** 1 Wisconsin National Primate Research Center, University of Wisconsin Graduate School, 1220 Capitol Court, Madison, WI 53715, USA; 2 Department of Pathology and Laboratory Medicine, University of Wisconsin Medical School, 600 Highland Avenue, Madison, WI 53792, USA; 3 Chakri Naruebodindra Medical Institute, Faculty of Medicine Ramathibodi Hospital, Mahidol University, Samut Prakan 10540, Thailand; 4 Departments of Statistics and of Biostatistics and Medical Informatics, Carbone Cancer Center, University of Wisconsin-Madison, Madison, WI, USA; 5 Morgridge Institute for Research, 330 N. Orchard Street, Madison, WI 53715, USA; 6 Department of Cell and Regenerative Biology, University of Wisconsin School of Medicine and Public Health, Madison, WI 53707-7365, USA; 7 Department of Molecular, Cellular, and Developmental Biology, University of California, Santa Barbara, Santa Barbara, CA 93106, USA

**Keywords:** Components of the immune system, Stem cells research, Developmental biology

## Abstract

Hemogenic endothelium (HE) is the main source of blood cells in the embryo. To improve blood manufacturing from human pluripotent stem cells (hPSCs), it is essential to define the molecular determinants that enhance HE specification and promote development of the desired blood lineage from HE. Here, using SOX18-inducible hPSCs, we revealed that SOX18 forced expression at the mesodermal stage, in contrast to its homolog SOX17, has minimal effects on arterial specification of HE, expression of HOXA genes and lymphoid differentiation. However, forced expression of SOX18 in HE during endothelial-to-hematopoietic transition (EHT) greatly increases NK versus T cell lineage commitment of hematopoietic progenitors (HPs) arising from HE predominantly expanding CD34^+^CD43^+^CD235a/CD41a^−^CD45^−^ multipotent HPs and altering the expression of genes related to T cell and Toll-like receptor signaling. These studies improve our understanding of lymphoid cell specification during EHT and provide a new tool for enhancing NK cell production from hPSCs for immunotherapies.

## Highlights

• SOX18-enforced expression in mesoderm does not affect arterial specification of HE
• Overexpression of SOX18 in HE leads to generation of NK versus T cell-biased progenitors
• SOX18 preferentially expands CD34^+^ progenitors with lin^−^CD43^+^CD45^−^ phenotype

## Graphical abstract

**Figure:**
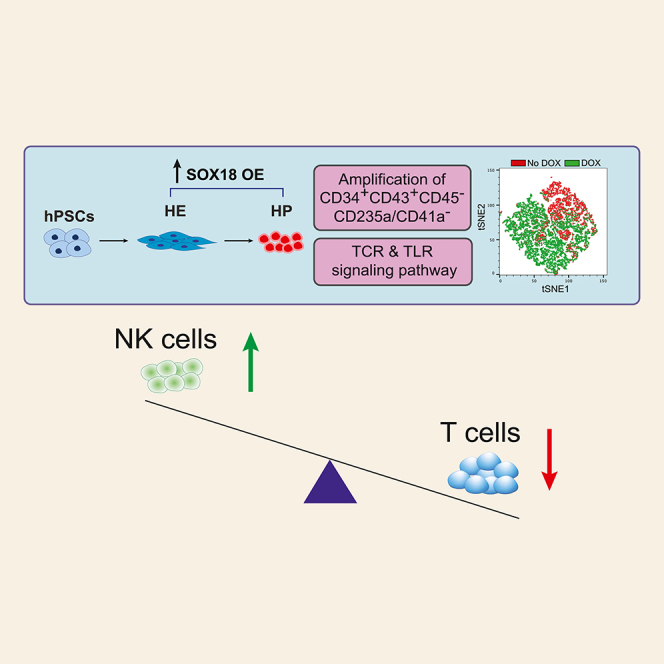

## Introduction

The SOXF family transcription factors, SOX7, SOX17 and SOX18, have been recognized as critical regulators of angiogenesis, cardiovascular and hematopoietic development.^1^^,^^2^^,^^3^^,^^4^^,^^5^^,^^6^^,^^7^^,^^8^ SOXF factors are expressed in hemogenic endothelium (HE) and clusters of hematopoietic cells emerging from HE.^3^^,^^4^^,^^6^^,^^9^^,^^10^ However, their expression is transient and not detected in terminally differentiated lymphoid and myeloid cells. Murine embryonic studies have shown that Sox7 is required for the formation of the earliest multipotent HPs with erythro-myeloid potential.^4^ Forced expression of Sox7 in cells from E7.5 mouse embryo or from *in vitro* differentiated mouse embryonic stem cells (ESCs) promotes self-renewal of early CD41^+^ hemogenic progenitors with erythro-myeloid potential and blocks their differentiation.^4^^,^^11^ A similar phenotype was observed following overexpression of Sox18 in *in vitro* differentiated mouse ESCs.^3^ In contrast, Sox17 is required for arterial specification,^12^ establishing the definitive, but not primitive, hematopoietic program,^10^ and maintaining intra-aortic hematopoietic clusters and fetal liver hematopoietic stem cells (HSCs).^5^^,^^6^^,^^7^ Using hPSCs, we demonstrated that SOX17 is a master regulator of HOXA and arterial programs in HE, and is required for the specification of HE with robust lympho-myeloid potential and DLL4^+^CXCR4^+^ phenotype resembling arterial HE at sites of HSC emergence.^13^ With activation of NOTCH signaling, SOX17 directly activates CDX2 expression leading to the upregulation of the *HOXA* cluster genes.^13^

Here, we investigated SOX18 effects on hematopoietic development from hPSCs. We demonstrated that enforced SOX18 expression has a limited effect on specification and diversification of HE but significantly affects NK versus T cell commitment when overexpressed in HE and during EHT. Specifically, SOX18 overexpression expands and greatly enhances NK cell potential of CD34^+^CD43^+^CD235a/CD41a^−^CD45^−^ multipotent hematopoietic progenitors (HPs), predominantly leading to the altered expression of genes related to T cell and Toll-like receptor signaling.

## Results

### SOX18-enforced expression enhances production of erythro-myeloid progenitors

**Figure 1:**
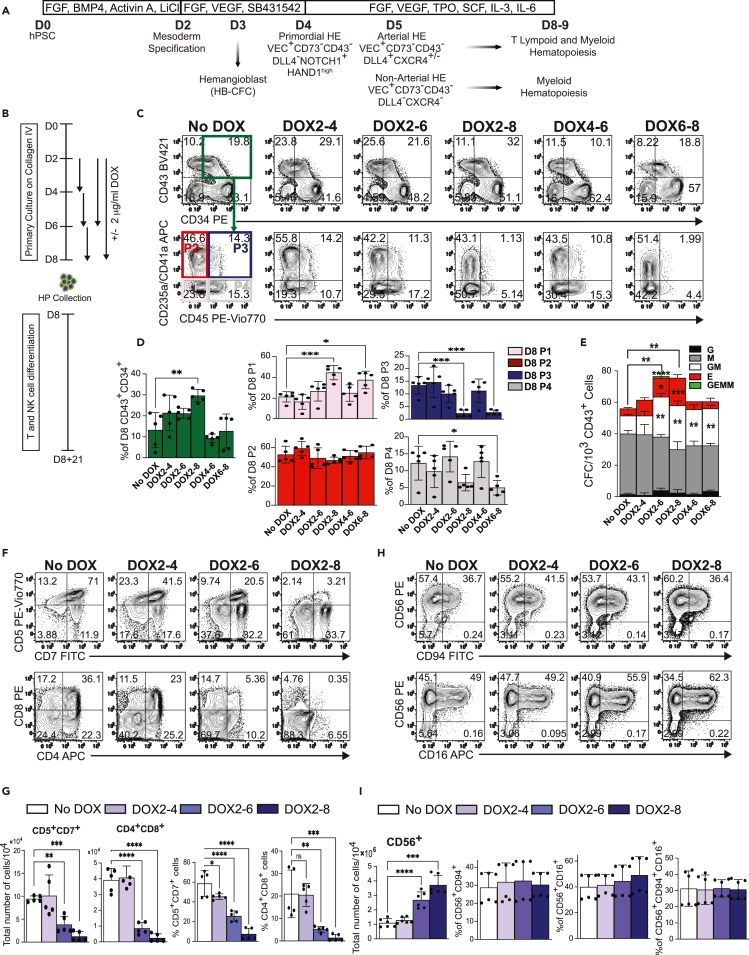
Effect of SOX18-enforced expression on hematopoietic differentiation of hPSCs (A) Schematic diagram of hematopoietic differentiation. D, day of differentiation. (B) Schematic diagram of experimental design. Cells were differentiated following DOX treatment as indicated. Floating HPs were collected on D8 of differentiation and evaluated for phenotype, CFC and lymphoid potential. (C) Representative flow cytometric counter plots show a phenotype hematopoietic progenitors isolated form D8 differentiation cultures of iSOX18 hPSCs. (D) Bar graphs show percentages of CD34^+^CD43^+^ cells and composition of CD43^+^CD34^+^ subsets on D8 of differentiation (results are mean ± SD, n = 5 for two independent experiments performed in triplicate and duplicate). ∗p<0.05, ∗∗p<0.01 and ∗∗∗p<0.001, one-way ANOVA, Tukey’s multiple comparisons test. (E) CFC potential of D8 HPs (results are mean ± SD, n = 2 experiments). ∗p<0.05, ∗∗p<0.01, ∗∗∗p<0.001, and ∗∗∗∗p<0.0001, two-way ANOVA. Significant differences for total CFCs are shown on the top of bars. Significant differences for each specific type of colony as compared to No DOX conditions are shown within the bar. (F) Flow cytometric analysis of T cell differentiation. (G) Total number of T cells generated from 10^4^ of D8 CD43^+^ cells and percentages of CD5^+^CD7^+^ and CD4^+^CD8^+^ cells (results are means ± SD, n = 5 experiments). ∗p<0.05, ∗∗p<0.01 ∗∗∗p<0.001, and ∗∗∗∗p<0.0001, one-way ANOVA, Dunnett’s multiple comparisons test. (H) Flow cytometric analysis of NK cell differentiation. (I) Graphs show the total number of CD56^+^ cells generated from 10^4^ CD43^+^ cells and the expression of CD94 and CD16 by NK cells (results are means ± SD, n = 6 for two independent experiments performed in triplicate). ∗p<0.05, ∗∗p<0.01, ∗∗∗p<0.001, and ∗∗∗∗p<0.0001, one-way ANOVA, Dunnett’s multiple comparisons test. See also Figures S1–S3.

To determine the impact of SOX18 overexpression on hematopoietic development in humans, we generated H1 hESCs carrying doxycycline (DOX)-inducible SOX18-P2A-Venus (Figure S1) and differentiated these cells under a chemically defined culture system in which all stages of hematopoietic development are temporally, phenotypically, and functionally defined (Figure 1A). In this differentiation system, the most primitive hemogenic cells with FGF2-dependent hemangioblast colony-forming cell (HB-CFCs) potential are detected on day 3 (D3) of differentiation.^14^^,^^15^^,^^16^ The first immature/primordial VEC^+^CD43^−^CD73^–^NOTCH1^+^ HE cells expressing high levels of *HAND1* mesodermal gene and lacking of arterial and venous gene expression arise on D4 (D4 HE). Subsequently on D5, HE specifies into DLL4^+^CXCR4^+/−^ arterial-type HE and DLL4^–^ non-arterial-type HE (Figure 1A).^14^^,^^16^^,^^17^^,^^18^ D5 DLL4^+^CXCR4^+^ HE is highly enriched in T lymphoid and multipotential myeloid progenitors and expresses the highest levels of *HOXA* and arterial genes, including *SOX17* and *NOTCH4*, as compare to other DLL4^+^ and DLL4^-^ HE populations.^13^ All hematopoietic progenitors derived from hPSC cultures on D8 of differentiation can be identified by CD43 expression.^19^^,^^20^ D8 CD34^+^CD43^+^ HPs are composed of at least three major subpopulations: (1) CD235a^+^CD41a^+^CD45^−^ progenitors enriched in erythro-megakaryocytic cells, (2) CD41a^lo^CD235a^+/−^CD45^+^ progenitors with erythro-myeloid potential, and (3) CD235a^−^CD41a^−^CD43^+^CD45^+/−^ multilineage progenitors that are lacking lineage markers, and display CD90^+^CD38^−^CD45RA^−^ phenotype^19^^,^^20^^,^^21^^,^^22^ typical for human hematopoietic stem/progenitor cells (HPSCs).^23^ In addition, it has been shown that SOX18 expression is initiated on D4 differentiation in HE and remains present in D5 HE and D8 HPs.^16^^,^^18^ To determine the stages of hematopoietic development sensitive to SOX18 modulation and the optimal duration of SOX18 overexpression to achieve a maximal effect on hematopoietic output, we treated hESC differentiation cultures with DOX at different time points and analyzed the phenotype and CFC potential of hematopoietic cells collected on D8 of differentiation (Figure 1B). As shown in Figures 1C and 1D, treatment of cultures with DOX from D2 through D8 (DOX2-8) resulted in the highest percentages of CD34^+^CD43^+^ HPs. We also noted that this treatment increased the proportion of CD235a/CD41a^−^CD45^−^ progenitors (D8 P1 population) within CD34^+^CD43^+^ population and decreased a proportion of CD235a/CD41a^+^CD45^+^ progenitors (D8 P3 population). Although a similar effect on subset composition within CD34^+^CD43^+^ progenitors was observed in DOX6-8, DOX treatments at these stages of differentiation had a minimal effect on the percentages of CD34^+^CD43^+^ cells. In contrast, earlier and shorter treatments (DOX2-4) mildly increased CD34^+^CD43^+^ cells, but had a minimal effect on their composition.

To visualize the in-depth phenotype of D8 hematopoietic cells from No DOX and DOX2-8 cultures, CD43^+^gated cells were analyzed using the t-distributed stochastic neighbor embedding algorithm (tSNE) which showed distinct single cell deposition between No DOX and DOX2-8 (Figure S2A). tSNE analysis also highlighted a suppression of CD45^+^ populations and enrichment of CD45^−^ and CD235a/41a^−^ populations following DOX treatment. In addition, DOX treatment reduced populations with CD235a/41a^hi/med^CD34^-^ phenotype (Figures S2B and S2C) corresponding to more mature erythro-myeloid cells. This observation is consistent with prior findings in murine system, which demonstrated a blocking effect of Sox18 on differentiation of ESC- and yolk sac-derived HPs.^3^

Analysis of CFCs revealed the most substantial effect of SOX18 upregulation in DOX2-6 and DOX2-8 cultures where we observed a significant increase in a frequency of GM- and E-CFCs (Figure 1E). Furthermore, we found that DOX treated cultures had a higher megakaryocytic differentiation potential than non-treated controls (Figures S3A and S3B). Overall, these findings suggests that SOX18 has the most profound effect on CD34^+^CD43^+^ HPs and GM- and E-CFCs when continuously upregulated from D2 through D8 of differentiation.

### SOX18-enforced expression suppresses T cell differentiation and promotes NK cell production

To evaluate the effect of SOX18 on lymphoid differentiation, we treated cultures with DOX as shown in Figure 1B and evaluated T cell and NK cell potential of HPs generated on day 8 of differentiation. Treatment of differentiation cultures with DOX suppressed T cell potential of day 8 HPs. This suppression of T cell potential was more pronounced in cultures treated with DOX from D2 through D8 (Figures 1F and 1G). Although we observed a minimal effect of SOX18-enforced expression on the proportion of NK cells and the expression of CD16 and CD94 by CD56^+^ cells, the total number of NK cells increased dramatically in cultures treated with DOX, especially following prolonged SOX18 upregulation during hematopoietic differentiation (DOX2-6 and DOX2-8 cultures; Figures 1F and 1G).

### SOX18-enforced expression promotes HB-CFCs but has a limited effect on specification of hemogenic endothelium formation

**Figure 2:**
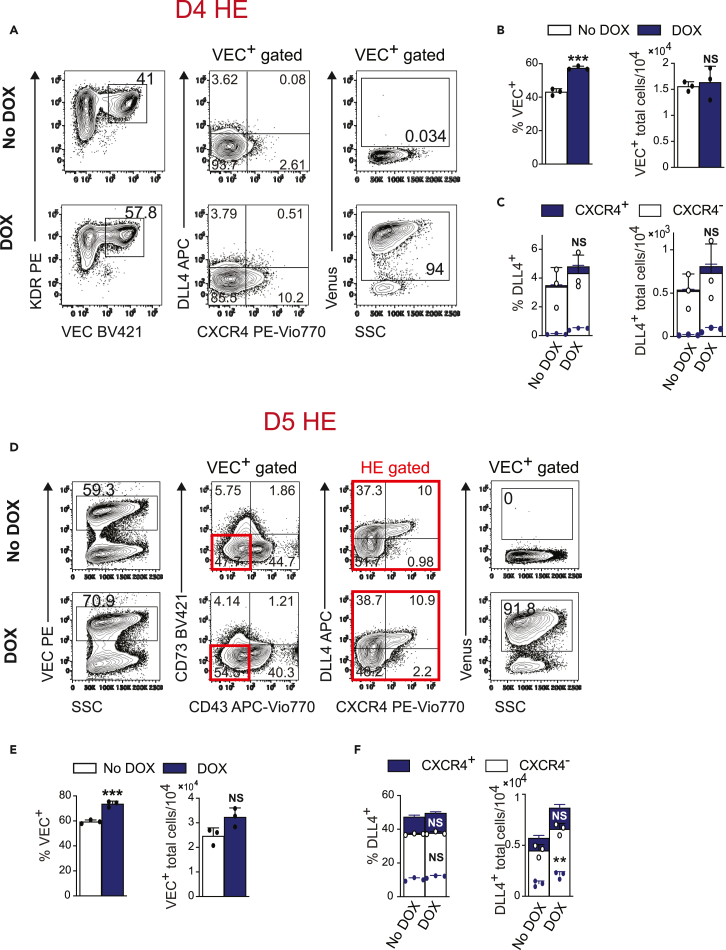
SOX18 has a limited effect on specification of HE (A) Expression of arterial markers and Venus in VEC^+^ iSOX18 cells on D4 of differentiation with or without DOX. (B and C) Percentages and total number of VEC^+^ cells, VEC^+^DLL4^+^CXCR4^+^ and VEC^+^DLL4^+^CXCR4^-^ cells generated from 10^4^ hPSCs on D4 of differentiation (results are mean ± SD, n = 3 experiments). ∗∗∗p<0.001, t-test. (D) Expression of arterial markers and Venus in VEC^+^ iSOX18 cells on D5 of differentiation with or without DOX. (E and F) Percentages and total number of VEC^+^ cells, VEC^+^DLL4^+^CXCR4^+^ and VEC^+^DLL4^+^CXCR4^-^ cells generated from 10^4^ hPSCs (results are mean ± SD, n = 3 experiments). ∗∗∗p<0.001, t-test, and ∗∗p<0.01, two-way ANOVA, Sidak’s multiple comparisons test.

To determine stages of hematopoiesis mostly affected by SOX18 overexpression, we analyzed the effect of DOX treatment on the formation of the HB colonies and HE, including arterial HE specification. We found that enforced expression of SOX18 on D2 of differentiation resulted in almost 3-fold increase in the numbers of HB colonies (Figure S3C). However, SOX18 overexpression had a limited effect on HE formation and arterial HE specification on D4 and D5 of differentiation (Figures 2A–2F). Although we noted a slight increase in the proportion of VE-cadherin^+^ (VEC^+^) endothelial cells in DOX-treated cultures, no significant differences were observed in the proportion of DLL4^+^CXCR4^+/−^ arterial HE in DOX and No DOX cultures. We also observed the formation of VEC^+^DLL4^−^CXCR4^+^ population in DOX-treated D4 cultures (Figure 2A). However, transcriptional profiling of DLL4^−^CXCR4^+^ and DLL4^−^CXCR4^-^ D4 VEC^+^ populations revealed no differentially expressed genes, including arterial genes (see “molecular characterization of SOX18-induced changes in HE and blood cells” section below).

**Figure 3:**
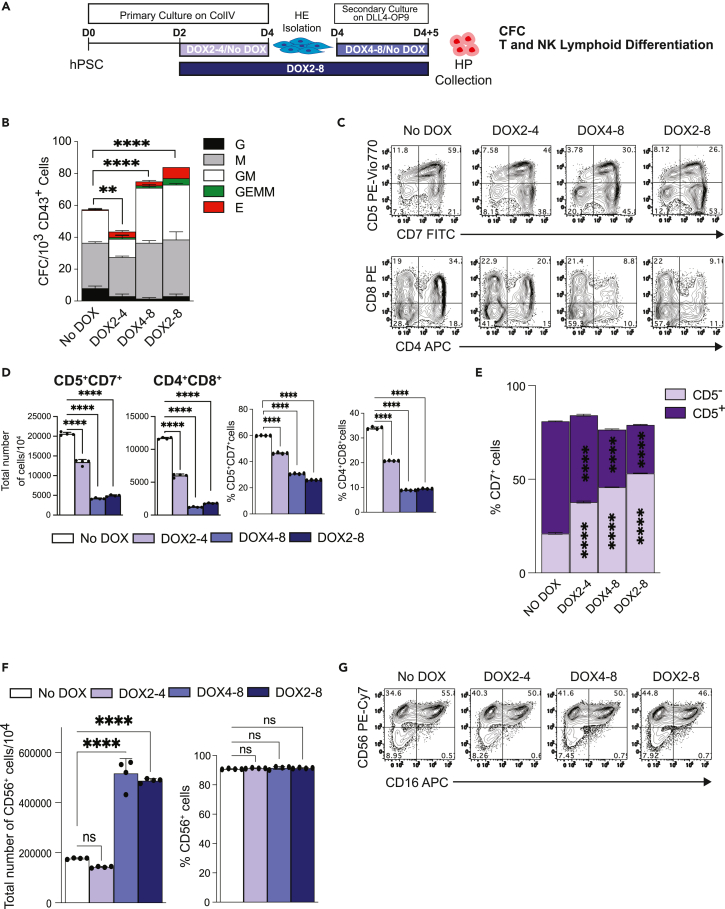
SOX18 overexpression affects lymphoid specification from HE by shifting the balance of NK versus T lymphocyte differentiation (A) Schematic diagram of experimental design. (B) CFC potential of HP generated from D4 HE after coculture on DLL4-OP9 for 5 days with or without DOX (results are mean ± SD, n = 2 experiments). ∗∗p<0.01 and ∗∗∗∗p<0.0001, two-way ANOVA, Tukey’s multiple comparisons test. (C) Representative dot plot showing T cell differentiation. (D) Total number and percentages of T cells generated from 10^4^ CD43^+^ cells (results are mean ± SD, n = 4 experiments). ∗∗∗∗p<0.0001, two-way ANOVA, Sidak’s multiple comparisons test. (E) Percentages of CD7^+^CD5^+/−^ cells generated in T cell cultures (results are mean ± SD, n = 4 experiments) ∗∗∗∗p<0.0001, two-way ANOVA, Tukey’s multiple comparisons test. (F) Flow cytometric analysis of NK cell differentiation. (G) Total number of CD56^+^ NK cells generated from 10^4^ CD43^+^ cells and percentages of CD56^+^ cells in NK differentiation cultures (results are mean ± SD, n = 4). ∗∗∗∗p<0.0001, two-way ANOVA, Sidak’s multiple comparisons test.

To assess SOX18 effect on HE, we isolated D4 HE generated in No DOX and DOX conditions and cultured on OP9-DLL4 in presence or absence of DOX (Figure 3A). Analysis of CFC potential revealed that treatment of HE with DOX (DOX4-8) or continuous treatment with DOX throughout culture (DOX2-8) significantly increased CFC formation whereas HE from primary differentiation cultures pretreated with DOX (DOX2-4) without additional DOX treatment during co-cultures with OP9-DLL4 generated fewer CFCs as compared to No DOX controls (Figure 3B).

Analysis of T cell potential revealed that HE from DOX2-4 treated cultures demonstrated decreased CD5^+^CD7^+^ and CD4^+^CD8^+^T cell output accompanied by increased in CD7^+^CD5^−^ cells. This effect was more pronounced when DOX treatment was initiated on D4 in co-culture of HE, or in DOX2-8 treatment cultures (Figures 3C–3E). In contrast, treatment of HE in OP9-DLL4 co-cultures markedly increased NK cell potential of D8 HPs, whereas D2-4 DOX treatment of primary differentiation cultures had no effect on NK cell output from HE (Figures 3F and 3G). These findings suggest that SOX18 overexpression predominantly affects lymphoid specification from HE by shifting the balance of NK versus T lymphocyte differentiation potential.

### SOX18-enforced expression promotes expansion of CD34^+^CD43^+^CD235a/CD41a^−^CD45^−^ (D8 P1) population enriched in NK cell potential

**Figure 4:**
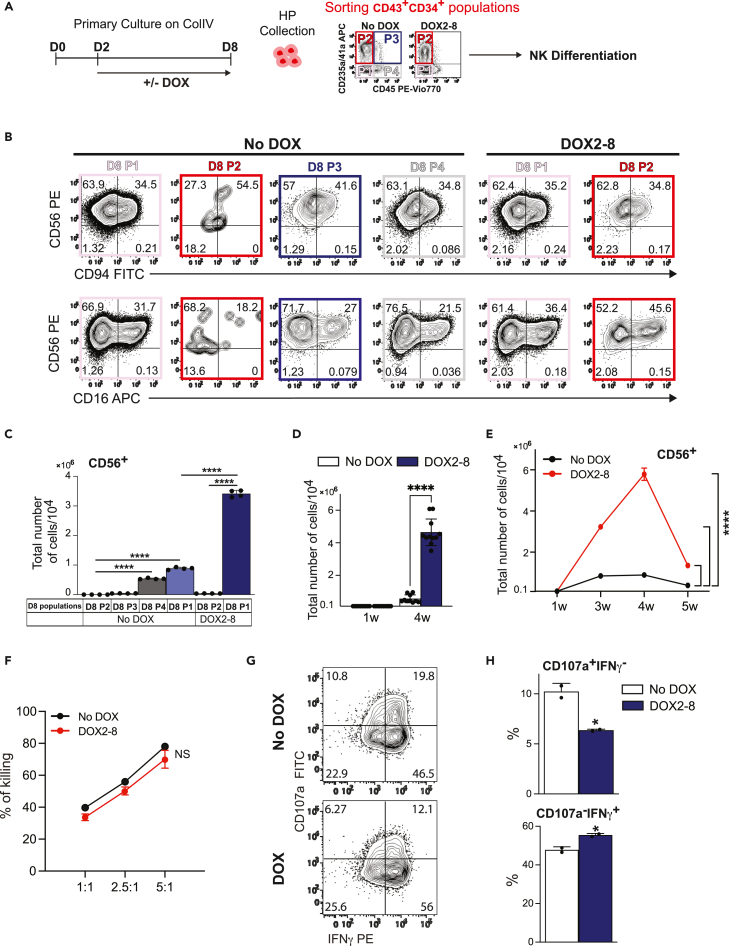
Characterization of NK cell potential of iSOX18 hPSCs (A) Schematic diagram of experimental design showing DOX treatment schedule and cells subsets analyzed. (B) Flow cytometric analysis of NK cell differentiation potential of cell subsets isolated from No DOX and DOX cultures. (C) Total number of CD56^+^ NK cells generated from 10^4^ CD43^+^ cells (results are means ± SDs, n = 4 experiments). ∗∗∗∗p<0.0001, one-way ANOVA, Tukey’s multiple comparisons test. (D) Total numbers of CD56^+^ cells in cultures from No DOX and DOX2-8 HP after 1 and 4 weeks differentiation in NK cultures (results are means ± SDs, n = 10–11). ∗∗∗∗p<0.0001, two-way ANOVA, Sidak’s multiple comparisons test. (E) Growth curve of CD56^+^ cells in NK differentiation cultures initiated from D8 CD43^+^ cells generated in No DOX and DOX2-8-treated cultures. Cell cultures were started with 10^5^ CD43^+^ cells and continued for 5 weeks (results are means ± SDs, n = 2). (F) Cytotoxicity assay against K562 targets. Different amount of target cells plated with CD56^+^ cells (effector: target ratio from 1:1 to 5:1) for 4 h (Results are means ± SDs, n = 2 experiments). (G and H) Flow cytometric counter plot shows CD107a and IFNγ expression by CD56^+^ cells after K562 stimulation and (H) Graphs show the percentages (results are means ± SDs, n = 2). ∗p<0.05 t-test. See also Figure S4.

As shown in Figures 1C and 1D, D2-D8 SOX18 overexpression significantly promoted development of CD235a/CD41a^−^CD45^−^ (D8 P1) populations and inhibited development of CD45^+^ populations, including CD235A/CD41a^+^ (D8 P3) and CD235a/CD41a^−^ (D8 P4) populations within CD34^+^CD43^+^ HPs. To define cell populations enriched in NK cells following DOX treatment, we isolated major populations of HPs formed on D8 of culture and assessed their NK cell potential (Figure 4A). As shown in Figures 4B and 4C, in control cultures, NK cell potential was mostly detected in D8 P1 and P4 populations, while P2 population failed to produce NK cells. Although CD235a/CD41a^+^CD45^+^ (D8 P3) cells were capable of producing NK cells (Figure S4A), the total NK cell output from this subset was negligible, as compared to the P1 and P4 populations (Figure 4C). Owing to dramatic inhibition of CD45^+^ cell development in DOX treated cultures, we evaluated the NK cell potential of only two major populations: (1) the D8 P1 population which markedly expanded following DOX treatment and (2) the D8 P2 population. These studies revealed that, in DOX-treated cultures, NK cell potential was detected in P1 and P2 populations (Figure 4B). However, total NK cell generation from P2 population was negligible (Figure 4C). Thus, we concluded that in DOX-treated cultures NK cell potential is mostly restricted to the D8 P1 population and that this population possesses much stronger NK cell differentiation potential as compared to the NK-producing populations in the control No DOX cultures. Overall, HPs collected from DOX2-8 treated cultures generate up to 5-fold more NK cells as compared to the control (Figure 4D). Moreover, kinetic analysis of NK differentiation cultures revealed a more rapid and longer expansion of NK cells generated from HPs in DOX-treated differentiation cultures (Figure 4E). Functional assessment of NK cells revealed no substantial differences in cytotoxic potential, IFNγ production or degranulation response against K562 cells of NK cells generated from DOX and No DOX conditions (Figures 4F–4H). Overall, these studies indicate that enforced SOX18 expression predominantly expands CD34^+^CD43^+^CD235a/CD41a^−^CD45^−^ (D8 P1) HP population with superior NK cell potential.

### Molecular characterization of SOX18-induced changes in HE and blood cells

**Figure 5:**
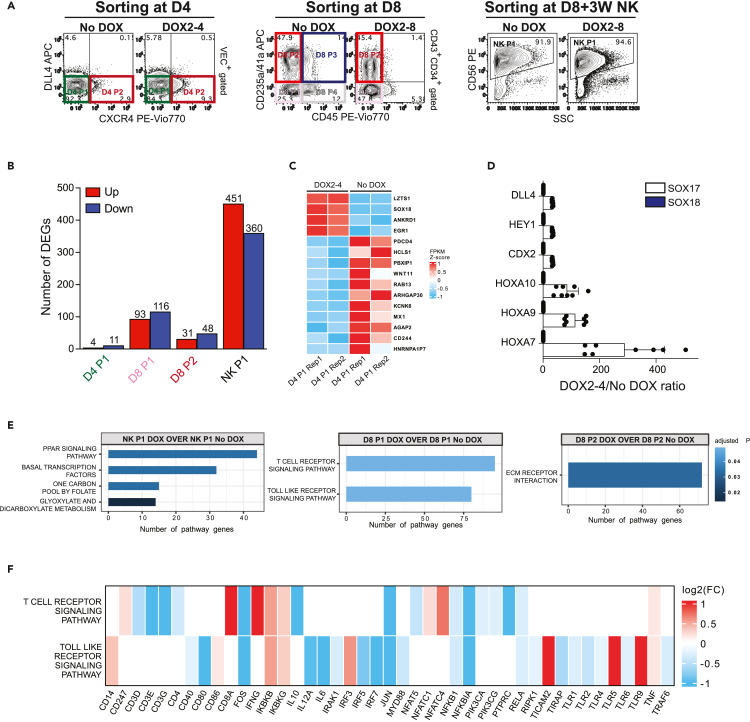
Molecular profiling of HE, HPs and NK cells generated from iSOX18 hPSCs (A) Counter plots showing cell populations isolated for RNA-seq analysis. D4 plots shows cell subsets within VEC^+^ gated cells and D8 plots show cell subsets within CD34^+^CD43^+^ gated cells. (B) Graph shows the number of DEGs in indicated populations. (C) Heatmap shows differentially expressed genes in D4 HE P1 population from cultures with and without DOX. (D) qRT-PCR analysis compares the expression of indicated genes in D4 HE generated from iSOX18 and iSOX17 hPSCs following D2-4 DOX treatment (results are mean ± SD, n = 8). (E) Bar plot shows significantly enriched KEGG pathways in selected differential expression comparisons. Y axis shows the enriched pathway categories and x axis shows the number of significantly genes in each pathway. (F) Fold change of selected genes in the two KEGG pathways that are significantly enriched in D8 P1 DOX+ cells over D8 P1 No DOX cells. See also Figure S5.

To define changes in transcriptional program induced by forced SOX18 expression, we performed RNA-seq analysis of D4 HE, D8 CD34^+^CD43^+^ subsets and NK cells generated from No DOX and DOX-treated cultures (Figure 5A). We found only 15 differentially expressed genes (DEGs) in the D4 DLL4^−^CXCR4^-^ VEC^+^ population (D4 P1 population) following D2-4 DOX treatment (Figures 5B and 5C and Data S1), suggesting minimal effect of SOX18 on D4 HE. In contrast to our prior findings of D2-4 SOX17 overexpression,^13^ RNA-seq analysis of SOX18 D4 HE from No DOX and DOX cultures showed no significant increase in expression of *HOXA* genes, *CDX2*, or genes involved in NOTCH signaling pathways. These findings were confirmed using qPCR analysis of selected genes in D4 CD31^+^ HE cells isolated from control and D2-4 DOX-treated differentiation cultures of iSOX17^13^ and iSOX18 hPSCs (Figure 5D). Because our flow cytometric analysis showed that SOX18 overexpression induces DLL4^−^CXCR4^+^ subset within D4 HE (Figures 2A and 5A), we analyzed DEGs in DLL4^−^CXCR4^+^ (D4 P1) and DLL4^−^CXCR4^-^ (D4 P2) populations from DOX-treated cultures. These studies revealed no DEGs in these subsets, including expression of arterial genes, thus suggesting that CXCR4 expression in D4 HE without co-expression of DLL4 does not reflect activation of arterial program.

The most significant changes in transcriptional programs were observed in the D8 P1 multipotent HP population and in CD56^+^ NK cells which showed 209 and 811 DEGs in their corresponding DOX treated and untreated cultures (Figure 5B and Data S1). Gene set enrichment analysis (GSEA) in D8 P1 cells revealed enrichment in Kyoto Encyclopedia of Genes and Genomes (KEGG) categories related to T cell receptor (TCR) and Toll-like receptor (TLR) signaling pathways (Figure 5E), suggesting that SOX18 overexpression has a major effect on establishing T lymphoid transcriptional program in the D8 P1 multipotent HPs arising from HE. As shown in Figure 5F, downregulated genes in these categories included CD3 complex genes, *CD4*, AP1 complex genes *JUN* and *FOS*, *PI3K* signaling genes and the majority of TLR genes, whereas upregulated genes included *IFNG*, *TICAM2*, *TLR5* and *TLR9*. D8 P2 population in DOX+ and DOX-conditions showed only 79 DEGs enriched in extracellular matrix (ECM) receptor interactions KEGG categories (Figure 5E and Data S1). GSEA in NK cells generated from DOX treated and untreated cultures demonstrated enrichment in KEGG categories related to glyoxylate and dicarboxylate metabolism, one carbon pool by folate, and PPAR signaling pathway (Figure 5E and Data S1). However, no differential expression of NK cell activating receptors *KLRK1, NCR2, NCR1* or key NK cell transcription factors *EOMES* and *TBX21* was observed between NK cells from DOX-treated and untreated cultures (Data S1). These findings indicate that overexpression of SOX18 during EHT has a major impact on the transcriptional program regulating metabolism of NK cells.

To determine whether SOX18 overexpression affects proliferation and apoptosis in major cell subsets, we performed gene sets enrichment analysis in GO categories related to “Regulation of Cell Population Proliferation” and “Apoptosis”. We found that the first GO gene set was significantly altered in D4 and D8 P1 subsets and NK cells in DOX+ versus No DOX comparisons, whereas apoptotic genes were mostly affected in D8 P1 subset (Figures S5A and S5B). To confirm the effect of SOX18 on cell proliferation, we performed cell-cycle analysis of D5 differentiated cells using bromodeoxyuridine (BrdU). Consistent with gene expression analysis, SOX18 overexpression led to a significant cell-cycle shift from G0/G1 to S phases in HE cells and emerging CD43^+^ HPs (Figures S5A and S5B).

## Discussion

Previous studies using inducible murine ESCs or embryos revealed that SOX18 overexpression during the early stages of hematopoietic development enhances the development of blast colonies composed of immature HPs.^3^ Herein, we investigated how SOX18 overexpression affects specification and diversification of HE from hPSCs. Using an inducible hPSC line, we demonstrated that SOX18 overexpression during the mesodermal stage of differentiation has little effect on HE specification, including establishment of arterial phenotype. However, SOX18 exerted a profound effect on the development of NK cells versus T cells when upregulated in HE and during EHT. We also inferred that SOX18-enforced expression promotes the development of HB-CFCs and erythro-myeloid progenitors, including megakaryocytes. Overexpression of SOX18 downregulates expression of CD45 within hPSC-derived CD34^+^CD43^+^ HPs and promotes generation of CD34^+^CD43^+^CD235a/CD41a^−^CD45^−^ cells (D8 P1 population) enriched in NK cell potential. Molecular profiling of D8 P1 populations generated with and without DOX treatment showed that SOX18 overexpression has the most significant impact on genes involved in TCR and TLR signaling pathways. These findings suggest that SOX18 has a major effect on specification and lineage commitment of HPs arising from HE.

In addition to their role in hematopoietic development, SOXF group factors including SOX17 and SOX18 are involved in cardiovascular development, where they function in redundant mode.^1^^,^^24^^,^^25^ Our current SOX18 and previous SOX17 studies^13^ suggest that the function of these transcription factors in human hematopoietic development is likely distinct and non-redundant. Although SOX17 has the most profound effect on specification of DLL4^+^CXCR4^+^ arterial HE with T lymphoid potential,^13^ SOX18 demonstrated no effect on development and formation of arterial HE. However, SOX18 affected NK versus T lymphoid potential of hemogenic progenitors when overexpressed in already established HE. Molecular profiling studies revealed that in contrast to SOX17,^13^ SOX18 overexpression at the mesodermal stage of development caused very limited changes in the gene expression profile of HE and did not affect arterial, NOTCH signaling, *CDX2* or *HOXA* cluster gene expression in HE. In contrast, SOX18 overexpression during EHT caused the most significant changes in transcriptional program of multipotent HPs arising from HE, mostly affecting genes involved in TCR and TLR signaling.

Hematopoietic development in the vertebrate embryo occurs in multiple waves. The first transient wave of hematopoiesis takes place from HB in the yolk sac blood islands giving rise to primitive erythroid, megakaryocytic and macrophage cells that are different from their corresponding adult counterparts. In contrast to the first wave of primitive hematopoiesis lacking of lymphoid and granulocytic potential, subsequent waves of definitive hematopoiesis produce the entire spectrum of adult-type erythro-myeloid progenitors (EMPs), lymphomyeloid progenitors, and finally HSCs with the capacity for long-term repopulation of an adult recipient.^26^^,^^27^^,^^28^^,^^29^ However, it became evident that EMPs can also produce NK cells and γ/δ T cells arising from CD7^+^CD5^−^ lymphoid progenitors.^30^^,^^31^^,^^32^ Recent studies demonstrated that in hPSC cultures, NK cells originate from at least two independent CD34^+^ hemogenic progenitors which can be discriminated based on HOXA expression. HOXA^low/−^CD34^+^ progenitors produce NK cells enriched in genes associated with innate immune response, expressing higher levels of CD16 and displaying more robust cytotoxic and degranulation potential, whereas HOXA^+^CD34^+^ progenitors generate NK cells enriched in genes associated with inflammatory responses and greater IFNγ production.^32^ Our studies revealed that SOX18-enforced expression had no effect on proportion of CD16^+^ or CD94^+^ cells within CD56^+^ NK cell population and did not affect cytotoxic potential of NK cells against K562 targets or expression of genes associated with innate immune response or inflammation. These observations suggest that SOX18 does not cause a preferential expansion of NK population with distinct degranulation versus pro-inflammatory responses.

NK cell therapies combined with genetic engineering technologies have emerged as increasingly powerful tools for immunotherapies of cancers. The use of hPSCs as an unlimited source of NK cells can further expand the applicability of cellular immunotherapies by offering “off-the-shelf” therapeutic products to fit specific clinical needs for a broad group of patients.^33^^,^^34^ Our finding that SOX18 promotes development of HPs with superior NK cell potential, whereas inhibiting T cell development, will advance our comprehension of molecular network that regulates specification of lymphoid cells from HE. This understanding will help to improve the scalability of NK cell production from hPSCs for immunotherapies.

### Limitations of the study

Although these studies revealed a distinct and contrasting effect of SOX18 overexpression, as compared to SOX17 overexpression, on specification and gene expression in HE, HPs, and lymphoid cells from hPSCs, the molecular mechanisms mediating this effect remains to be addressed. We have shown an increased number of CD34^+^CD43^+^CD235a/CD41a^−^CD45^−^ HPs enriched in NK cell potential following SOX18 overexpression. Subsequent studies will define whether SOX18 is required for development of this population or NK cell specification.

## STAR★Methods

### Key resources table

**Table:** 

REAGENT or RESOURCE	SOURCE	IDENTIFIER
**Antibodies**		

Annexin V-APC	BD Biosciences	Cat# 550474; RRID: AB_2868885
CD4 APC (clone: RPA-T4)	BD Biosciences	Cat# 555349; RRID: AB_2033930
CD5 PE-Vio770 (clone: REA782)	Miltenyi Biotec	Cat# 130-111-109; RRID: AB_2658600
CD7 FITC (clone: M-T701)	BD Biosciences	Cat# 555360; RRID: AB_395763
CD8 PE (clone: HIT8a)	BD Biosciences	Cat# 555635; RRID: AB_395997
CD16 APC	BD Biosciences	Cat# 561248; RRID: AB_10612010
CD31 MicroBeads	Miltenyi Biotec	Cat# 130-091-935
CD34 PE (clone: 581)	BD Biosciences	Cat# 555822; RRID: AB_396151
CD41a APC (clone: HIP8)	BD Biosciences	Cat# 559777; RRID: AB_398671
CD41a PerCP-Cy5.5 (clone: HIP8)	BD Biosciences	Cat# 340930; RRID: AB_400183
CD42b PE (clone: HIP1)	BD Biosciences	Cat# 555473; RRID: AB_395865
CD43 APC-Vio770 (clone: DF-T1)	Miltenyi Biotec	Cat# 130-101-174; RRID: AB_2658135
CD43 BV421 (clone: 1G10)	BD Biosciences	Cat# 562916; RRID: AB_2737890
CD43 BV510 (clone: 1G10)	BD Biosceinces	Cat# 563377; RRID: AB_2722767
CD45 PE-Vio770 (clone: 5B1)	Miltenyi Biotec	Cat# 130-113-119; RRID: AB_2725947
CD56 PE (clone: B159)	BD Biosciences	Cat# 555516; RRID: AB_395906
CD56 PE-Cy7 (clone: B159)	BD Biosciences	Cat# 560916; RRID: AB_2033963
CD61 APC (clone: VI-PL2)	BD Biosciences	Cat# 564174; RRID: AB_2738645
CD73 BV421 (clone: AD2)	BD Biosciences	Cat# 562430; RRID: AB_11153119
CD73 PE-Vio770 (clone: AD2)	Miltenyi Biotec	Cat# 130-120-795; RRID: AB_2752200
CD94 FITC (clone: HP-3D9)	BD Biosciences	Cat# 555888; RRID: AB_396200
CD107a FITC (clone: H4A3)	BD Biosciences	Cat# 555800; RRID: AB_396134
CD144 BV421 (clone: 55-7H1)	BD Biosciences	Cat# 565670; RRID: AB_2744284
CD144 PE (clone: REA199)	Miltenyi Biotec	Cat# 130-118-495; RRID: AB_2751528
CD184 PE-Vio770 (clone: REA649)	Miltenyi Biotec	Cat# 130-120-797; RRID: AB_2857538
CD235a APC (clone: GA-R2 (HIR2))	BD Biosceinces	Cat# 551336; RRID: AB_398499
CD309 PE (clone: 89106)	BD Biosciences	Cat# 560494; RRID: AB_1645503
DLL4 APC (clone: MHD4-46)	Miltenyi Biotec	Cat# 130-096-560; RRID: AB_10827749
Ghost Dye Violet 540	TONBO Biosciences	Cat# 13-0879-T100
GAPDH (clone: FL335)	Santa Cruz Biotechnology	Cat# SC-47724; RRID: AB_627678
Human SOX18	R&D	Cat# AF5077; RRID: AB_2195670
IFN ***γ*** PE (clone: B27)	BD Biosciences	Cat# 559327; RRID: AB_3397224
Rabbit IgG HRP	Santa Cruz Biotechnology	Cat# SC-2357; RRID: AB_628497
Sheet IgG HRP	R&D	Cat# HAF016; RRID: AB_562591
7AAD	BD Pharmingen	Cat# 559925; RRID: AB_2869266

**Chemicals, peptides, and recombinant proteins**		

Recombinant Human/Murine/Rat Activin A	PeproTech	Cat# 120-14E
Recombinant Human BMP4	PeproTech	Cat# 120-05ET
Recombinant Human FGF-basic	PeproTech	Cat# 100-18B
Recombinant Human VEGF_165_	PeproTech	Cat# 100-20
Recombinant Human IL-6	PeproTech	Cat# 200-06
Recombinant Human IL-3	PeproTech	Cat# 200-03
Recombinant Human SCF	PeproTech	Cat# 300-07
Recombinant Human TPO	PeproTech	Cat# 300-18
Recombinant Human Flt3-Ligand	PeproTech	Cat# 300-19
Recombinant Human IL-7	PeproTech	Cat# 200-07
Recombinant Human IL-11	PeproTech	Cat# 200-11
Recombinant Human IL-15	PeproTech	Cat# 200-15
LiCl	Sigma	Cat# L9659
SB431542 (TGF-β inhibitor)	Cayman Chemical	Cat# 13031
Collagen IV	Sigma	Cat# C5533
Y-27632	Cayman Chemical	Cat# 10005583
Doxycycline hyclate	Sigma	Cat# D9891
Cell activation cocktail (without BrefeldinA)	BioLegend	Cat# 423301
Brefeldin A	ThermoFisher	Cat# 00-4506-51

**Critical commercial assays**		

BrdU KIT	BD Pharmingen	Cat# 552598
Human stem cell nucleofector kit2	Lonza	Cat# VPH-5022

**Deposited data**		

RNA-seq	This study	GEO: GSE195670

**Experimental models: Cell lines**		

K562-GFP	This paper	N/A
pTRE-SOX18-P2A-Venus-rpEF1a-Zeo and pEF1α-M2rtTA-T2A-Puro (PiggyBac) H1 hESC line	This paper	N/A

**Oligonucleotides**		

CDX2 Forward	Sigma-Aldrich	CCGAACAGGGACTTGTTTAGAG
CDX2 Reverse	Sigma-Aldrich	AGGTTGGCTCTGGCATTTATAG
DLL4 Forward	Sigma-Aldrich	CAGTGGGCAGCGAAGCTACA
DLL4 Reverse	Sigma-Aldrich	ACAGGCAGTGGTAGCCATCCTC
HEY1 Forward	Sigma-Aldrich	GGACTATCGGAGTTTGGGATTT
HEY1 Reverse	Sigma-Aldrich	TGGGAAGCGTAGTTGTTGAG
HOXA7 Forward	Sigma-Aldrich	AGGTCCAGGATCAGGGTATT
HOXA7 Reverse	Sigma-Aldrich	CCAGAGAAGGAGGGATTGATTC
HOXA9 Forward	Sigma-Aldrich	GCGCCTTCTCTGAAAACAAT
HOXA9 Reverse	Sigma-Aldrich	CAGTTCCAGGGTCTGGTGTT
HOXA10 Forward	Sigma-Aldrich	GCAAAGAGTGGTCGGAAGAA
HOXA10 Reverse	Sigma-Aldrich	CGCTCTCGAGTAAGGTACATATTG
SOX18 Forward	Sigma-Aldrich	GGCAAAGCGTGGAAGGA
SOX18 Reverse	Sigma-Aldrich	CGGCCGGTACTTGTAGTTG
RPL13A Forward	Sigma-Aldrich	CCTGGAGGAGAAGAGGAAAGAGA
RPL13A Reverse	Sigma-Aldrich	TTGAGGACCTCTGTGTATTTGTCAA

**Recombinant DNA**		

pTRE-SOX18-P2A-Venus-rpEF1α-Zeo	This paper	N/A
pEF1α-M2rtTA-T2A-Puro	This paper	N/A
Super piggyBac transposase expression vector	Transposagen	Cat# SPB-DNA

**Software and algorithms**		

Prism versions 9	GraphPad Software Inc.	https://www.graphpad.com/scientific-software/prism/
FlowJo 8.8.6	FlowJo	https://www.flowjo.com/
FlowJo 10	FlowJo	https://www.flowjo.com/
STAR (version 2.5.2b)	(Dobin et al.^35^, PMID: 23104886)	https://github.com/alexdobin/STAR
RSEM (version 1.3.0)	(Li and Dewey^36^, PMID: 21816040)	https://deweylab.github.io/RSEM/
edgeR (version 3.34.1)	(Robinson et al.^37^, PMID: 19910308)	https://bioconductor.org/packages/release/bioc/html/edgeR.html
fgsea (version 1.18.0)	(Korotkevich et al.^38^, no PMID is available, https://doi.org/10.1101/060012)	https://bioconductor.org/packages/release/bioc/html/fgsea.html
ggplot2 (version 3.3.5)	Wickham. ggplot2: Elegant Graphics for Data Analysis. Springer-Verlag New York, 2016	https://ggplot2.tidyverse.org/
ComplexHeatmap (version 2.8.0)	(Gu et al.^39^, PMID: 27207943)	https://github.com/jokergoo/ComplexHeatmap

### Resource availability

#### Lead contact

Further information and requests for resources and reagents should be directed to and will be fulfilled by the lead contact, Igor I. Slukvin (islukvin@wisc.edu).

#### Materials availability

Plasmid and cell lines generated in this study will be made available on request. Transfer may require completion of material transfer agreement.

### Experimental model and subject details

#### Construction of vectors, generation and validation of iSOX18 hPSC line

A doxycycline (DOX)-inducible SOX18 H1 hESC line was generated using PiggyBac system.^40^ Human SOX18 CDS was cloned into PiggyBac transposon vector (Transposagen) downstream of TREtight promoter of pTRE-P2A-Venus-rpEF1a-Zeo plasmid, and co-transfected with pEF1α-M2rtTA-T2A-Puro and transposase plasmid into H1 cells using human stem cell nucleofector kit 2 (Lonza). Cells were selected in Zeocin (0.5 μg/mL, Thermofisher) and Puromycin (0.5 μg/mL, Sigma) for 10 days and resistant clones were screened by Venus expression with DOX (Sigma) treatment.

### Method details

#### hPSC maintenance and hematopoietic differentiation

hPSCs were maintained and passaged on Matrigel in mTeSR1 media (WiCell). Hematopoietic differentiation was performed on collagen IV (ColIV)-coated plates in chemically defined serum-free medium as described.^14^ The iSOX18 H1 line from hPSCs (H1 hESC line from WiCell) was maintained and passaged on Matrigel in mTeSR1 media (WiCell). The cell lines were differentiated on a collagen IV (ColIV)-coated plate.^14^ To initiate differentiation, cells were plated at 5,000 cells/cm^2^ onto 6 well plates with E8 media and 10 μM Rock inhibitor (Y-27632, Cayman Chemicals). This media was changed the following day to IF9S media with 50 ng/mL FGF2 (PeproTech), 50 ng/mL BMP4 (PeproTech), 15 ng/mL Activin A (PeproTech), and 2 mM LiCl (Sigma), and cells were cultured in hypoxia (5% CO_2_, 5% O_2_). On day 2, the media was changed to IF9S media with 50 ng/mL FGF2, 50 ng/mL VEGF (PeproTech), and 2.5 μM TGF-β inhibitor (SB-431542, Cayman), and cells were cultured in hypoxia (5% CO_2_, 5% O_2_). On days 4 and 6, the media was changed to IF9S media with 50 ng/mL FGF2, 50 ng/mL VEGF, 50 ng/mL TPO (PeproTech), 50 ng/mL IL-6 (PeproTech), 20 ng/mL SCF (PeproTech), and 10 ng/mL IL-3 (PeproTech), and cells were cultured in normoxia (20% CO_2_, 5% O_2_).

#### Isolation and culture of hemogenic endothelium and hematopoietic progenitors

HE from D4 cultures was isolated by magnetic activated cell sorting (MACS) using CD31 antibodies. In D4 differentiation cultures almost all CD31^+^ cells co-express VE-cadherin.^16^ Selected cells were plated on OP9 or DLL4-OP9 in 10% α-MEM with 10% FBS (Hyclone) with TPO (50 ng/mL), SCF (50 ng/mL), IL-6 (20 ng/mL), IL-3 (10 ng/mL) and FLT3L (10 ng/mL). To overexpress SOX18, cells were cultured with or without 2 μg/mL DOX. Medium was changed on the following day and extra medium was added on the 3^rd^ day of OP9 coculture. After 5 days in secondary culture, cells were collected and assessed for colony-forming cells (CFCs), T, and NK potential. HPs generated from iSOX18 cells were collected at day 8 (D8) of differentiation. Four different subsets of CD34^+^CD43^+^ population CD235a/CD41a^−^CD45^−^ (P1 subset), CD235a/CD41a^+^CD45^−^ (P2), CD235a/CD41a^+^CD45^+^ (P3) and CD235a/CD41a^−^CD45^+^ (P4) were isolated using an MA900 cell sorter (Sony Biotechnology) and cultured on OP9-DLL4 in NK cell differentiation conditions.

#### T-cell differentiation

For T cell differentiation, floating hematopoietic cells from D8 of primary differentiation cultures or day 4+5 secondary OP9-DLL4-cocultures were cultured in α-MEM (Invitrogen) with 20% FBS (Hyclone), 10 ng/mL SCF, 5 ng/mL FLT3L and 5 ng/mL IL-7 (PeproTech) on OP9-DLL4 for 3 weeks. Cells were passaged weekly onto fresh OP9-DLL4 cells. Cells were analyzed by flow cytometry for T cell surface markers after 21 days. All cytokines were purchased from PeproTech.

#### NK-cell differentiation

For NK cell differentiation, floating hematopoietic cells from D8 primary cultures or day 4+5 secondary OP9-DLL4-cocultures were cultured in α-MEM (Invitrogen) with 20% FBS (Hyclone), 25 ng/mL SCF, 5 ng/mL FLT3L, 5 ng/mL IL-3 and 5 ng/mL IL-7 and 10 ng/mL IL-15 (PeproTech) on OP9-DLL4 for 5 days. Cells were then cultured in the same media without IL-3 for 3–4 weeks. Cells were passaged weekly onto fresh DLL4-OP9 cells and analyzed by flow cytometry for NK cell surface markers after 3–4 weeks.

#### Hemangioblast (HB)-CFC and hematopoietic CFC assay

HB-CFCs were detected using a semisolid colony-forming serum-free medium (CF-SFM) containing 40% ES-Cult M3120 methylcellulose (2.5% solution in IMDM, Stem Cell Technologies), 25% StemSpan serum-free expansion medium (SFEM, Stem Cell Technologies), 25% human endothelial serum-free medium (ESFM, ThermoFisher), 10% BIT 9500 supplement (Stem Cell Technologies), GlutaMAX (1/100 dilution, ThermoFisher), Ex-Cyte (1/1000 dilution, Millipore), 100 mM MTG, 50 mg/mL ascorbic acid and 20 ng/mL FGF (Peprotech).^15^ Hematopoietic CFCs were detected using serum containing H4435 MethoCult (Stem Cell Technologies).

#### Megakaryocyte differentiation

Floating hematopoietic cells from iSOX18 hPSC cultures were collected at D8 of differentiation and cultured in StemSpan serum-free expansion medium (SFEM, Stem Cell Technologies) with 20 ng/mL SCF, TPO, and IL-11 on an ultra-low attachment 6- well plate for 5 days. Fresh media (2 mL) was added every 2 days. All cytokines were purchased from PeproTech.

#### Functional analysis of NK cells

To asses cytotoxicity, CD56^+^ cells were isolated using an MA900 cell sorter and incubated with K562-GFP target cells for 4 h at 37°C, at effector:target (E:T) ratio of 1:1, 2.5:1, and 5:1, in 96 well plate. Cells were collected in FACS buffer, and stained 7-Aminoactinomycin D (7-AAD) and Annexin V (BD). Specific killing was calculated by subtracting spontaneous K562 death (7-AAD+ cells in no effector control). Production of IFN***γ*** and CD107a expression in isolated CD56^+^ cells was assessed after incubation with PMA and ionomyocin (1:500) (BioLegend) for 4 hours. Brefeldin A (1:1000; Thermo Fisher) was added at the beginning of the stimulation. Cells were washed with FACS buffer and stained with Live/dead violet 540 (TONBO bioscience) and CD107a antibody (BD). Cells were treated with fixation/permeabilization buffer (eBioscience) and stained for intracellular IFN***γ*** (BD).

#### Flow cytometry and tSNE analysis

Flow cytometric analysis was performed using antibodies listed in key resources table, MACSQuant Analyzer 10 (Miltenyi Biotech) and FlowJo 10 software (FlowJo LLC). No FITC-conjugated antibodies are used for analysis of D2-D8 differentiated cells to avoid overlap with Venus signal in FL1 channel. However, panel for phenotypic characterization of NK cells included FITC-conjugated antibodies since expression of Venus is completely downregulated after 1 week of HP culture in NK differentiation conditions (Figure S4B).

For tSNE analysis, individual DOX2-8 and No DOX fcs files were imported into FlowJo to exclude doublets, debris and dead cells. A subset or 11,000 CD43^+^ cells were selected for each sample and concatenated. To generate a tSNE map, the concatenated data were analyzed with the parameters 30 perplexity, 550 iteration number and 1540 learning rate (Eta). Concatenated cells were divided manually into No DOX or DOX2-8 and cell subsets were defined by the manual gating. First, cells CD34^+^ or CD34^−^ were gated and then gated CD235a/CD41a^hi^CD45^-^, CD235a/CD41a^med^CD45^-^, CD235a/CD41a^−^CD45^−^, CD235a/CD41a^−^CD45^+^, CD235a/CD41a^+^CD45^+^ cells.

#### Apoptosis and cell cycle analysis

Apoptosis was detected by flow cytometry using Annexin V (BD). For cell-cycle analysis, D5 cells were incubated in culture medium with BrdU (10 μM, BD Pharmingen) for 2 hours and stained with antibodies. For BrdU detection, the BrdU flow kit with 7 AAD was used and performed per the manufacturer’s instructions. Fluorescent reagents used for analysis, cell viability, apoptosis, and proliferation are listed in key resources table.

#### Real time qPCR

RNA was extracted from D4 CD31^+^ cells isolated from control and DOX-treated cultures of iSOX18 of iSOX17^13^ hPSCs using the RNeasy Plus MicroKit (QIAGEN). RNA was reverse-transcribed into cDNA using random hexamer primers (QIAGEN) with SMART MMLV reverse transcriptase (TaKaRa). qPCR was conducted using TB Green Advantage qPCR Premix (TaKaRa). RPL13A was used as the reference gene to normalize the data. Primer sequences are listed in key resources table.

#### RNA-seq

One hundred nanograms of total RNA was used to prepare sequencing libraries following the Ligation Mediated Sequencing (LM-Seq) protocol^41^ and quantified with a Qubit fluorometer (Life Technologies). Final cDNA libraries were quantitated with the Quant-iT PicoGreen Assay Kit (ThermoFisher Scientific), multiplexed, loaded at a final concentration of 1 nM or 2.5 nM, and sequenced as single reads on the NextSeq 2000 (Illumina), respectively.

#### Western Blot

For Western Blot experiment, iSOX18 hPSCs were cultured with (2 μg/ml) and without DOX for 24 hours and harvested. In a similar manner, total cells from iSOX18 hPSC differentiation cultures with (D2-D5) and without DOX were collected at day 5 of differentiation. The cells were lysed using Pierce IP lysis buffer with Pierce protease inhibitors. For cell lysate analysis, protein levels were quantified using the Pierce BCA Assay kit (Thermo Fisher, Waltham, MA) and normalized to 8 μg of total protein (depending on the individual blot) prior to running on pre-cast 4–12% gradient SDS-PAGE gels and subsequent transfer to PVDF membranes using Bio-Rad Trans-Blot Turbo Transfer System. The membrane was blocked with 5% BSA (Fisher Scientific, BP1600-100) and 5% Difco^TM^ Skim Milk (BD, 232100) in TBST (1%) for human SOX18 antibody (R&D Systems, 1:1000) and anti-GAPDH (Santa Cruz Biotechnology, 1:5000) probing respectively. The membranes were incubated with primary antibodies overnight at 4°C after blocking with mild agitation. The membranes were blotted with their corresponding HRP-linked secondary antibodies at room temperature for one hour. The antibodies were diluted in 1% BSA and 1% milk in TBST for SOX18 and GAPDH detection respectively. 1% TBST was used to wash the membranes for three times at 5 minutes intervals. Sheep and rabbit HRP-linked secondary antibodies were purchased from R&D Systems and Santa Cruz Biotechnology. Images were collected using Bio-Rad ChemiDoc^TM^ XRS+.

### Quantification and statistical analysis

#### RNA-seq analysis

RNA-seq reads were aligned by STAR (version 2.5.2b) to the human genome (version hg38) with GENCODE basic gene annotations (version 38). Gene expression levels were quantified by RSEM (version 1.3.0), and differential expression was analyzed by edgeR (version 3.34.1). A differentially expressed gene was required to have at least two-fold changes and an adjusted p-value < 0.05. Gene set enrichment analysis was performed by fgsea (version 1.18.0) with gene sets from the Molecular Signatures Database (version 7.1).

#### Statistical analysis

Data were analyzed using GraphPad Prism version 9 (GraphPad Software Inc.) and Microsoft Excel (Microsoft Corporation). Tests for statistical significance are listed with each experiment; these included two-sided Student’s t-tests for paired analyses and one way ANOVAs, and two way ANOVAs for experiments with multiple comparisons of variables or grouped variables, accompanied by the Tukey and Sidak post-hoc test, as inferred to be most appropriate by the software.

### Additional resources

Accession codes: The RNA-seqdata have been deposited to the Gene Expression Omnibus under accession GSE195670.

## Data Availability

• RNA-seq data have been deposited at GEO and are publicly available as of the date of publication. Accession numbers are listed in the key resources table. Figure S1 includes original western blot images. • This paper does not report the original code. • Any additional information required to reanalyze the data reported in this paper is available from the lead contact upon request.

## Supplemental information

Document S1. Figures S1–S5

Data S1. Differentially expressed genes in hemogenic subsets and NK cells generated from hPSC with and without SOX18-enforced expression, related to Figure 5
