# Supplementary material for: SOX18-enforced expression diverts hemogenic endothelium-derived progenitors from T towards NK lymphoid pathways
Source: iScience. 2023 Apr 8;26(5):106621. doi: 10.1016/j.isci.2023.106621 (PMC10214392; doi:10.1016/j.isci.2023.106621)
Supplement: Document S1. Figures S1–S5 [file mmc1.pdf]

iScience, Volume *26*

Supplemental information

SOX18-enforced expression diverts hemogenic endothelium-derived progenitors from T towards NK lymphoid pathways

Ho Sun Jung, Kran Suknuntha, Yun Hee Kim, Peng Liu, Samuel T. Dettle, Divine Mensah Sedzro, Portia R. Smith, James A. Thomson, Irene M. Ong, and Igor I. Slukvin

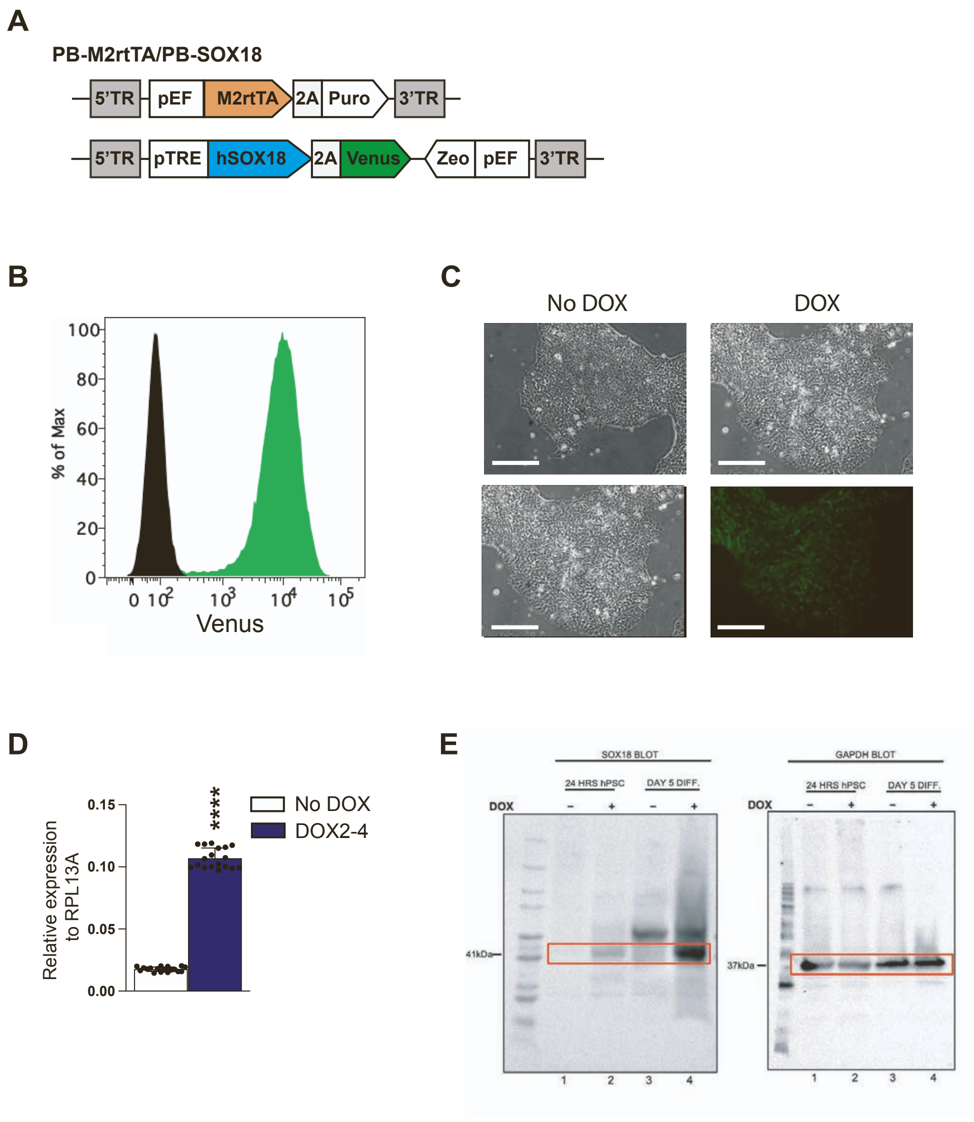

Supplementary Figure S1. Related to Figure 1. Generation of DOX-inducible SOX18 in H1 hESC line. (A) Schematic diagram of PiggyBac system used to generate iSOX18 cells. (B) Flow cytometric analysis and (C) fluorescent images show the expression of Venus reporter in undifferentiated iSOX18 cells cultured with or without DOX. Scale bars are 200 μm. (D) qRT-PCR analysis shows SOX18 expression in D4 HE generated in iSOX18 hPSCs differentiation cultures with or without DOX. Results are mean ± SD (n=9). ****p<0.0001, t-test. (E) Western blot shows upregulation of SOX18 expression in undifferentiated iSOX18 cells 24 hours after DOX treatment and D5 differentiated iSOX18 hPSCs treated with DOX at D2-D5.

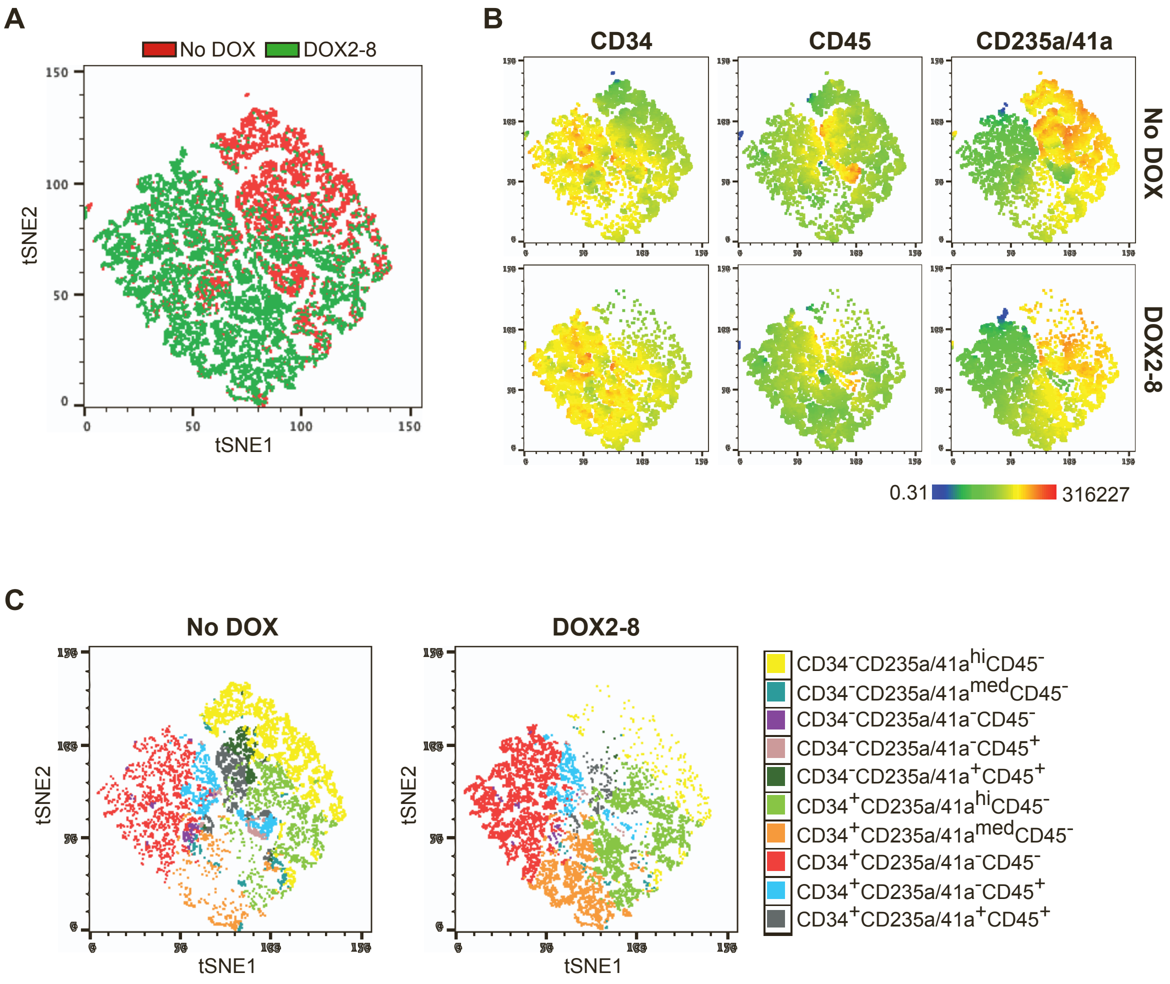

Supplementary Figure S2. Related to Figure 1. In-depth analysis of D8 $CD43^+$ subsets collected from cultures without and with D2-8 DOX using t-SNE. (A) Overlayed t-SNE plot of No DOX and DOX2-8 $CD43^+$ subsets at D8. Each dot represents one cell. (B) No DOX or DOX2-8 samples create single tSNE with single surface marker in each plot. Expression level of marker defines with a blue-green-yellow-red continuous color scale. (C) tSNE maps generated for the No DOX or DOX2-8 subsets. Each color indicates No DOX or DOX2-8 cells at D8 $CD43^+$. Each dot represents one cell and cells are colored according to their assigned subsets. Cell populations defined by the manual gating strategy.

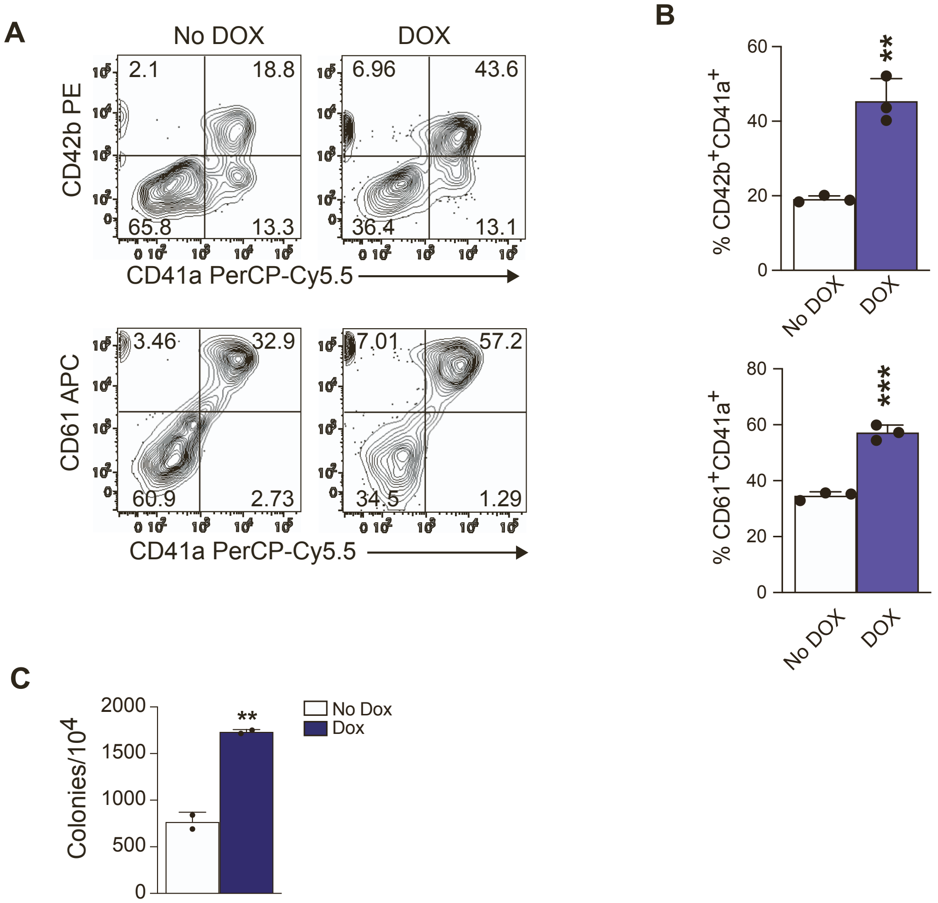

Supplementary Figure S3. Related to Figure 1. The effect of SOX18 overexpression on development of megakaryocytes and hemangioblasts. (A) Representative counter plot shows expression megakaryocytic markers in megakaryocyte differentiation cultures initiated using $CD43^{+}$ cells collected from No DOX and DOX2-8 cultures at D8. (B) Percentage of megakaryocytic cells generated in these cultures. Results are means ± SDs, n = 3 experiments; ** p<0.01 and ***p<0.001, t-test. (C) HB-CFC potential of iSOX18 cells. Cells collected from D3 differentiation without DOX or with D2-3 DOX treatment. Graph shows HB colonies per 10^4 cells collected on D3 of differentiation. Results are mean ± SD, n=2. **p<0.01, t-test.

A

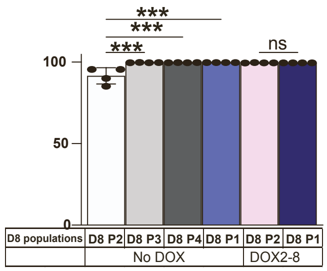

B

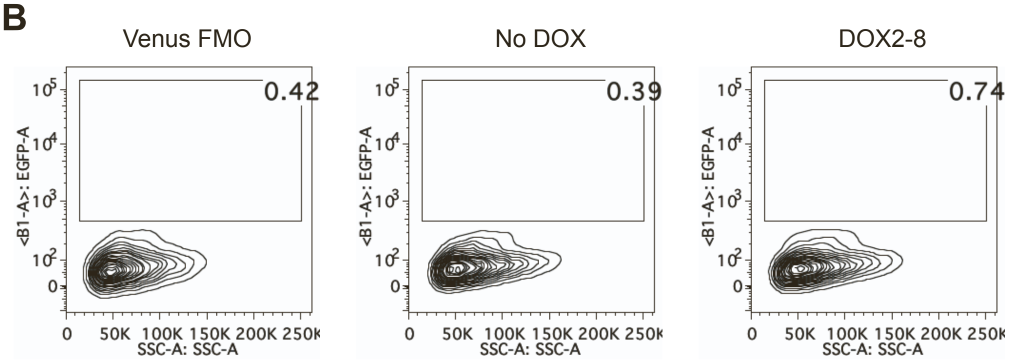

Supplementary Figure S4. Related to Figure 4. The effect of SOX18 enforced expression on NK cell differentiation potential. (A) Percentages of $CD56^+$ cells in NK cell differentiation cultures initiated from indicated subsets of hematopoietic progenitors generated in No DOX and DOX-treated conditions (results are means ± SDs, n=4 experiments; ***p<0.001). (B) Venus expression is completely downregulated in NK cell differentiation cultures.

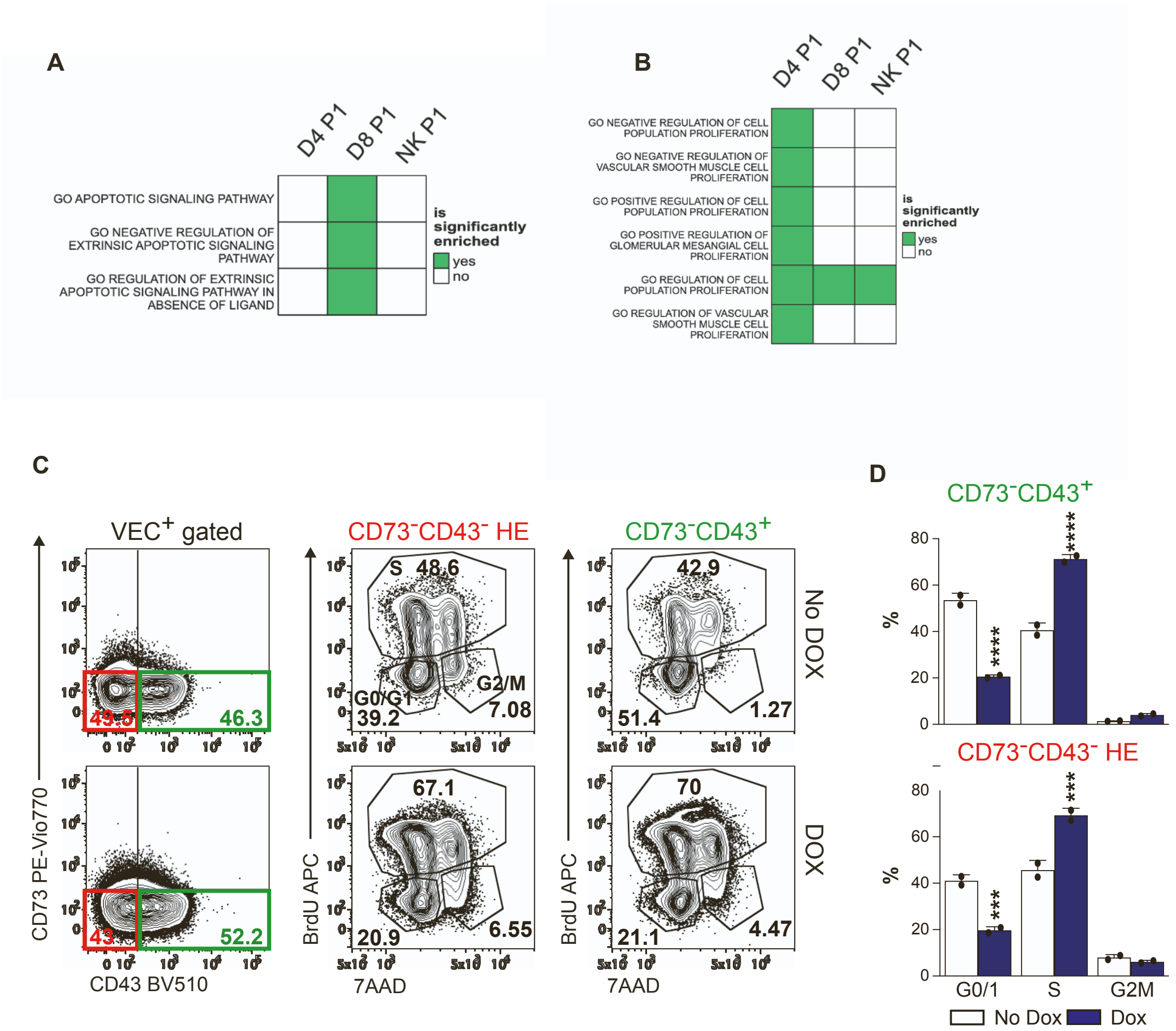

Supplementary Figure S5. Related to Figure 5. The impact of SOX18 overexpression on cell cycle and apoptosis. (A) Analysis of gene enrichment in GO “Apoptosis” and (B) “Regulation of Cell Population Proliferation” gene sets in indicated cell subsets in DOX-treated and untreated cultures. Green color indicates significant enrichment in DOX-treated over untreated cultures. (C) and (D) Flow cytometric analysis of cell cycle in the D5 HE in DOX and No Dox iSOX18 hPSC cultures. Representative dot plots (C) and mean ± SD (D) of duplicated experiments are shown. ***p<0.001, and ****p<0.0001, one-way ANOVA Dunnett’s multiple comparisons test.
